# Characterizing IgG4-related disease with ^18^F-FDG PET/CT: a prospective cohort study

**DOI:** 10.1007/s00259-014-2729-3

**Published:** 2014-04-25

**Authors:** Jingjing Zhang, Hua Chen, Yanru Ma, Yu Xiao, Na Niu, Wei Lin, Xinwei Wang, Zhiyong Liang, Fengchun Zhang, Fang Li, Wen Zhang, Zhaohui Zhu

**Affiliations:** 1 Department of Nuclear Medicine, Peking Union Medical College Hospital, Chinese Academy of Medical Sciences and Peking Union Medical College, No. 1 Shuaifuyuan, Wangfujing Street, Dongcheng District, Beijing, 100730 China; 2 Department of Rheumatology, Peking Union Medical College Hospital, Chinese Academy of Medical Sciences and Peking Union Medical College, No. 1 Shuaifuyuan, Wangfujing Street, Dongcheng District, Beijing, 100730 China; 3 Department of Pathology, Peking Union Medical College Hospital, Chinese Academy of Medical Sciences and Peking Union Medical College, Beijing, 100730 China

**Keywords:** IgG4-related disease, FDG PET/CT, Steroid-based therapy, Immune-mediated inflammation

## Abstract

**Purpose:**

IgG4-related disease (IgG4-RD) is an increasingly recognized clinicopathological disorder with immune-mediated inflammatory lesions mimicking malignancies. A cohort study was prospectively designed to investigate the value of ^18^F-fluorodeoxyglucose (FDG) positron emission tomography/computed tomography (PET/CT) in characterizing IgG4-RD.

**Methods:**

Thirty-five patients diagnosed with IgG4-RD according to the consensus criteria were enrolled with informed consent. All patients underwent baseline ^18^F-FDG PET/CT evaluation. Among them, 29 patients underwent a second ^18^F-FDG PET/CT scan after 2 to 4 weeks of steroid-based therapy.

**Results:**

All 35 patients were found with ^18^F-FDG-avid hypermetabolic lesion(s); 97.1 % (34/35) of these patients showed multi-organ involvement. Among the 35 patients, 71.4 % (25/35) patients were found with more organ involvement on ^18^F-FDG PET/CT than conventional evaluations including physical examination, ultrasonography, and computed tomography (CT). ^18^F-FDG PET/CT demonstrated specific image characteristics and pattern of IgG4-RD, including diffusely elevated ^18^F-FDG uptake in the pancreas and salivary glands, patchy lesions in the retroperitoneal region and vascular wall, and multi-organ involvement that cannot be interpreted as metastasis. Comprehensive understanding of all involvement aided the biopsy-site selection in seven patients and the recanalization of ureteral obstruction in five patients. After 2 to 4 weeks of steroid-based therapy at 40 mg to 50 mg prednisone per day, 72.4 % (21/29) of the patients showed complete remission, whereas the others exhibited > 81.8 % decrease in ^18^F-FDG uptake.

**Conclusion:**

F-FDG PET/CT is a useful tool for assessing organ involvement, monitoring therapeutic response, and guiding interventional treatment of IgG4-RD. The image pattern is suggested to be updated into the consensus diagnostic criteria for IgG4-RD.

## Introduction

IgG4-related disease (IgG4-RD) has been an increasingly recognized clinical entity in recent years [1–3]. As an immune-mediated inflammatory disease characterized by swelling lesions with storiform fibrosis and lymphoplasmacytic infiltration enriched with IgG4-positive plasma cells, IgG4-RD have been found in multiple organs/tissues, including the pancreas (also known as autoimmune pancreatitis, AIP), pancreatobiliary tract, lacrimal gland, salivary gland, lung, retroperitoneal region, and kidney [4]. Although several molecular and cellular mechanisms have been found to be correlated with IgG4-RD, the etiology of the disease is still unknown [5, 6]. AIP is a major and well-studied constituent of IgG4-RD. In 2003, multiple extrapancreatic lesions with rich infiltration of IgG4-positive plasma cells were reported; these lesions are similar to the pancreatic lesions in patients with AIP [7, 8]. Since then, many medical conditions, such as sclerosing sialadenitis, retroperitoneal fibrosis, and lymphoplasmacytic aortitis, were subsequently reported to be associated with AIP, indicating that these diseases may be part of a spectrum of clinical entities not yet well recognized [9–11]. A new clinicopathological disorder called IgG4-related disease was then proposed [12, 13]. However, many details about the gradually recognized clinical entity remain unclear. Inadequate understanding may cause misdiagnosis of the disease as malignancies. Such misunderstanding can cause high psychological pressure in the patients, excessive examinations, and even unnecessary surgical intervention when the condition can actually be cured by corticosteroid-based treatment [14, 15].

Integrated positron emission tomography and computed tomography (PET/CT) with ^18^F-fluorodeoxyglucose (^18^F-FDG) as the tracer provides metabolic information on the entire body; ^18^F-FDG PET/CT has been broadly used in the diagnosis, staging, response evaluation, and relapse monitoring of various types of malignancies [16]. ^18^F-FDG PET/CT is also used to assess both infectious and non-infectious inflammatory conditions [17]. A few recent case reports and retrospective analysis have preliminarily indicated possible value of ^18^F-FDG PET/CT in IgG4-RD evaluation [18–25]. A prospective cohort study was then designed to investigate the efficacy of ^18^F-FDG PET/CT in characterizing this tumor-like but benign disease and in evaluating IgG4-RD’s response to steroid-based treatment.

## Patients and methods

### Patients

This study was approved by the Institute Review Board of Peking Union Medical College Hospital (S-442) and registered online at the NIH ClinicalTrial.gov (NCT01665196). From May 2011 to December 2012, a total of 35 patients (M 23, F 12, aged 57 ± 12 years), mainly presented with Mikulicz’s disease (14/35, 40.0 %), autoimmune pancreatitis (12/35, 34.3 %), and retroperitoneal fibrosis (8/35, 22.9 %), were recruited with written informed consent. All patients were finally diagnosed with IgG4-RD according to the 2011 consensus criteria of IgG4-RD [26, 27]. The serum IgG4 level ranged from 163 mg/dl to 6,860 mg/dl (mean ± standard deviation: 1,649 ± 1,756, normal < 135). Twenty-one (60.0 %) patients presented with allergies. Twenty (57.1 %) patients had a pathological diagnosis. The demographics of the patients are listed in Table 1.

**Table 1:** Demographics of the patients with IgG4-related disease

No.	Age (year)/ Gender	Findings and diagnosis before ^18^F-FDG PET/CT	Additional involvement detected by ^18^F-FDG PET/CT	Serum IgG4 (mg/dl)	Biopsy site	Treatment	Follow-up evaluation
1	65/M	Interstitial nephritis, Prostatitis	Aorta	423	Kidney	Prednisone	Yes
2	66/M	Retroperitoneal fibrosis, lymphadenopathy	Kidney, more lymph nodes, lung, prostate	1,040	Lymph node	Prednisone	Yes
3	73/M	Autoimmune pancreatitis, sclerosing cholangitis	Salivary glands, lymph nodes, aorta	3,710	None	Prednisone, cyclophosphamide, methotrexate	Yes
4	61/M	Autoimmune pancreatitis, inflammatory pseudotumour	None	183	None	Prednisone, T2^a^, Tamoxifen	Yes
5	64/F	Autoimmune pancreatitis	Lymph nodes, salivary gland, lung	1,770	Submandibular gland	Prednisone, azathioprine	Yes
6	58/F	Mikulicz’s disease, lymphadenopathy	More lymph nodes, aorta	1,270	Lymph node	Prednisone, cyclophosphamide	Yes
7	30/F	Mikulicz’s disease, lymphadenopathy	None	163	Lymph node	Prednisone, cyclophosphamide	Yes
8	69/M	Autoimmune pancreatitis, lymphadenopathy	Liver, lymph nodes, prostate	3,520	None	Prednisone, cyclophosphamide	Yes
9	56/M	Autoimmune pancreatitis	Lymph nodes	319	None	Prednisone	Yes
10	74/F	Autoimmune pancreatitis, sclerosing cholangitis	None	331	None	Prednisone	Yes
11	57/M	Mikulicz’s disease	Lung, liver, bile duct, lymph nodes, retroperitoneal fibrosis, aorta, prostate	2,470	Submandibular gland	Prednisone	Yes
12	56/M	Mikulicz’s disease, lung lesions, prostatitis, lymphadenopathy	Retroperitoneal fibrosis, pleura	1,370	Parotid	Prednisone, cyclophosphamide	Yes
13	58/M	Retroperitoneal fibrosis	None	696	None	Prednisone	Yes
14	23/M	Mikulicz’s disease	Lymph nodes	402	Lymph node	Prednisone, cyclophosphamide	Yes
15	44/M	Mikulicz’s disease, lung lesions, lymphadenopathy	None	2,770	Lymph node	Prednisone, cyclophosphamide	Yes
16	69/M	Retroperitoneal fibrosis	Kidney, lymph nodes	403	None	Prednisone	Yes
17	53/F	Mikulicz’s disease, inflammatory pseudotumour	None	6,860	None	Prednisone, azathioprine	Yes
18	55/F	Mikulicz’s disease, lymphadenopathy	None	2,170	Submandibular gland, lymph node	Prednisone, cyclophosphamide	Yes
19	55/F	Retroperitoneal fibrosis	Salivary glands, lymph nodes, aorta	543	None	Prednisone, cyclophosphamide	Yes
20	55/M	Mikulicz’s disease, lymphadenopathy	Retroperitoneal fibrosis, pleura	5,630	Submandibular gland	Prednisone, cyclophosphamide	Yes
21	57/M	Autoimmune pancreatitis, sclerosing cholangitis, prostatitis	None	2,020	Pancreas	Prednisone, cyclophosphamide	Yes
22	47/M	Mikulicz’s disease, lymphadenopathy, pulmonary nodule	More lymph nodes	1,160	Lacrimal gland	Prednisone, azathioprine	Yes
23	55/F	Mikulicz’s disease, lymphadenopathy	Pancreas	986	None	Prednisone, cyclophosphamide	Yes
24	54/M	Mikulicz’s disease, lymphadenopathy	Pancreas, lung, liver, bile duct, kidney, prostate	3,910	Submandibular gland	Prednisone, cyclophosphamide	Yes
25	56/M	Retroperitoneal fibrosis	Lymph nodes, pancreas, aorta	356	Retroperitoneum	Prednisone, cyclophosphamide	Yes
26	71/M	Autoimmune pancreatitis, lymphadenopathy	Salivary gland, liver, biliary tract, prostate	2,180	None	Prednisone, cyclophosphamide	Yes
27	58/F	Retroperitoneal fibrosis, lymphadenopathy	Salivary gland, pancreas, more lymph nodes, kidney	394	Lymph node	Prednisone, methotrexate	Yes
28	58/M	Retroperitoneal fibrosis	Salivary gland, aorta, pancreas, lymph nodes, kidney	241	None	Prednisone, Tamoxifen	Yes
29	41/M	Mikulicz’s disease	Pancreas, lymph nodes, liver, spleen, cholangitis, retroperitoneal fibrosis, pericardium, pleura, prostate	6,030	Lymph node	Prednisone	Yes
30	74/M	Autoimmune pancreatitis, sclerosing cholangitis, prostatitis	Lymph nodes	423	None	Prednisone	No
31	73/M	Retroperitoneal fibrosis, Mikulicz’s disease	Lymph nodes, lung	501	Submandibular gland	Prednisone, mycophenolate mofetil	No
32	44/F	Autoimmune pancreatitis	Salivary glands, lymph nodes, aorta	903	None	Prednisone, cyclophosphamide	No
33	49/M	Autoimmune pancreatitis, lymphadenopathy	None	172	Pancreas	Prednisone, cyclophosphamide	No
34	47/F	Mikulicz’s disease, lymphadenopathy	None	1,000	Submandibular gland	Prednisone, methotrexate	No
35	55/F	Autoimmune pancreatitis	Salivary gland, lymph nodes	1,380	None	Prednisone	No

^a^T2: chloroform/methanol extract of *Tripterygium wilfordii* Hook F

### PET/CT scans

All patients were instructed to avoid strenuous work or exercise for at least 24 h, and to fast more than 4 h before intravenous injection of ^18^F-FDG at a dosage of 5.55 MBq (0.15 mCi) per kilogram body weight. The patients then rested in a warm, darkened room for nearly 1 h. After emptying the bladder, an acquisition was performed from mid-thigh to skull base (five to six bed positions, 2 min per bed) using a Siemens Biograph 64 Truepoint TrueV PET/CT scanner. Twenty-nine (82.9 %) patients also underwent a second PET/CT scan 2 to 4 weeks after the steroid-based treatments at a dosage of 40 mg to 50 mg prednisone per day (Table 1).

### Data analysis

The attenuation-corrected PET images and CT images were transferred to a Siemens MMWP multi-modality workstation. The visual analysis of PET/CT characteristics and pattern was done by three experienced nuclear medicine physicians and two rheumatologists through consensus reading using TrueD software (Siemens Medical Solution). The standardized uptake values (SUV) of the lesions were measured by the same nuclear medicine physician using the volume of interest (VOI) method and a unified standard. The images were transferred to an AW workstation (GE Healthcare) and then loaded onto a PET VCAR software (GE Healthcare) for response evaluation according to the PET Response Evaluation Criteria in Solid Tumor (PERCIST, version 1.0) [28]. The SUV corrected for lean body mass (SUL) and the total lesion glycolysis (TLG) were measured over the VOIs placed over the “hot” lesions of the involved organs or tissues. The changes in ^18^F-FDG uptake between the baseline and the follow-up scans were automatically calculated by the software, and finally reported as complete metabolic response, partial metabolic response, stable metabolic disease, or progressive metabolic disease according to the PERCIST 1.0.

### Statistical analysis

Continuous variables were summarized as means ± standard deviation; categorical variables were described in numbers and percentages. Continues variables between two groups were compared using student *t* test. The correlation between TLG and IgG4 level was evaluated by Pearson correlation coefficient. All tests were two-tailed with the significant level of 0.05. The analysis was carried out with the use of Prism 5.0.

## Results

### Assessing organ involvement

**Fig. 1:**
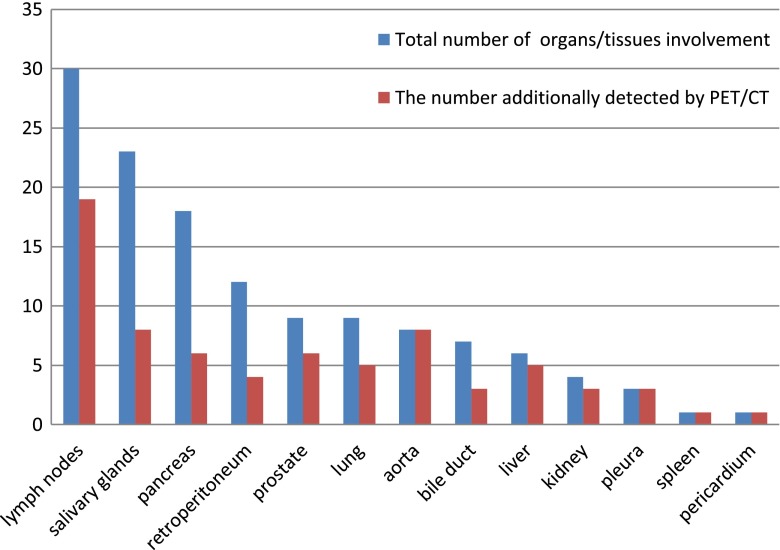
List of involved organs/tissues in the 35 IgG4-RD patients in descending order and the contribution of ^18^F-FDG PET/CT to the detection of involvement. *Blue bar*: total number of cases with the organs/tissues involved; *Red Bar*: the amount of involvement additionally detected by ^18^F-FDG PET/CT

All 35 patients were found with ^18^F-FDG-avid lesion(s) related to the disease. Thirty-four (97.1 %) patients showed lesions in more than one organ and 24 (68.6 %) had involvement in three or more organs. The most commonly involved organs were lymph nodes (30/35, 85.7 %), salivary glands (23/35, 65.7 %), and pancreas (18/35, 51.4 %). The involved organs or tissues are shown in Fig. 1 in descending order. In a total of 131 organ involvement in the 35 patients, ^18^F-FDG PET/CT additionally detected 40.5 % (53/131) organ involvement in 71.4 % (25/35) patients, including 100 % (8/8) of the aorta involvement, 63.3 % (19/30) of the lymph node involvement, 34.8 % (8/23) of the salivary gland involvement. Nine of ten patients enrolled with single organ disease were finally found with multi-organ involvement. Figure 1 illustrates the additional findings of ^18^F-FDG PET/CT in each organ, whereas the additional organ involvement demonstrated by ^18^F-FDG PET/CT in each patient is listed in Table 1.

### Characterizing image pattern

**Fig. 2:**
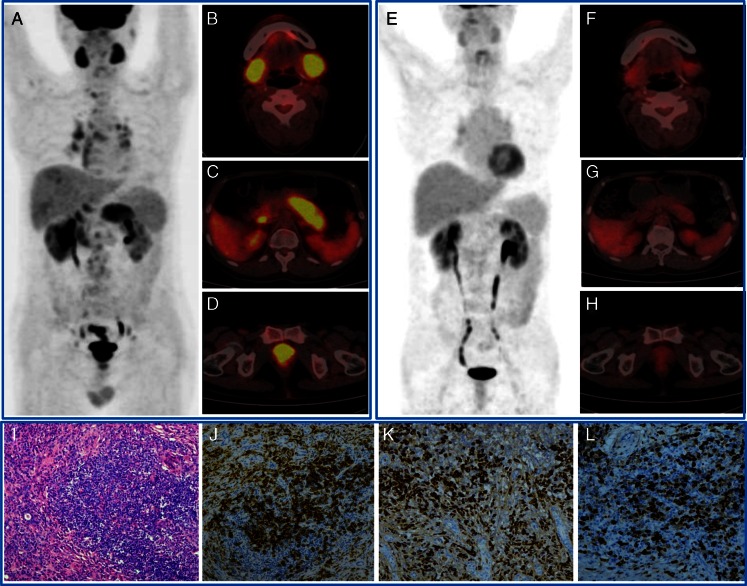
A 54-year-old man with IgG4-related disease showed multi-organ involvement (**a** whole-body view; **b** salivary glands; **c** pancreas; **d** prostate), significant response after two weeks of steroid-based treatment (**e**–**h**), and characteristic immunohistochemical stains of submandibular gland specimen (**i** HE stain; **j** CD38-positive plasma cells; **k** IgG-positive cells; **l** IgG4-positive cells. The IgG4-positive cells were > 60 % of the IgG-positive cells)

**Fig. 3:**
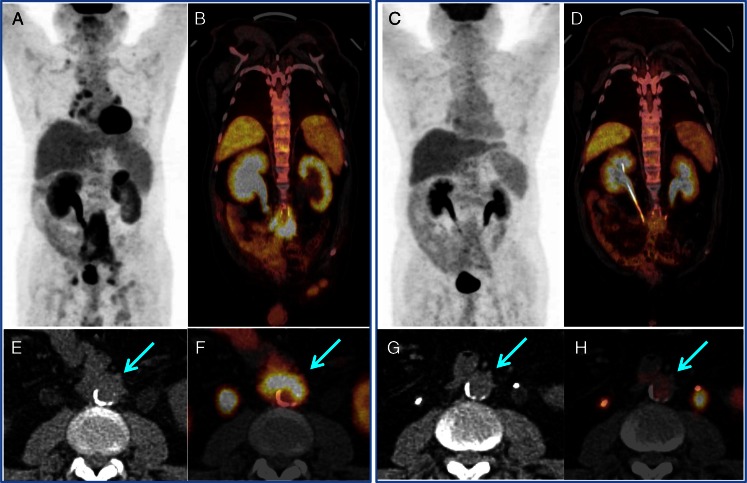
^18^F-FDG PET/CT guided the timely ureter recanalization in an IgG4-RD patient with retroperitoneal fibrosis and aorta involvement. The enlarged right renal pelvis with radioactive urine retention indicated severe ureteral obstruction, whereas the left side without radioactivity indicated complete obstruction (**a** and **b**). After D-J tube cannulation, the renal function was recovered bilaterally (**c** and **d**); the intense-uptake lesions were smaller and the intensity was significantly lower in response to the steroid treatment. The *arrows* show the aorta involvement beside the retroperitoneal fibrosis (**e**, **f**), which has a complete response to steroid-based treatment (**g**, **h**)

The lesions varied from diffuse infiltration to patchy or nodular forms, with the average SUV ranging from 1.1 to 8.3 (3.2 ± 1.4). Characteristic ^18^F-FDG distributions were observed in the PET/CT images of these patients. Table 2 summarizes the characteristics that we considered to be useful for indicating IgG4-RD rather than malignancies or other benign diseases. For each characteristic, the level of confidence for IgG4-RD indication was rated as strong, moderate, or weak according to our experiences in interpreting the images of IgG4-RD patients and our routine clinical patients, mainly by comparing with cancer patients. The most important characteristics were classified into four categories. The first category was diffusely elevated ^18^F-FDG uptake in the exocrine organ, such as salivary glands, pancreas, and prostate gland (Fig. 2). The second was patchy ^18^F-FDG-avid hypermetabolic lesions, mainly involving the retroperitoneal region, vascular wall, bile duct, lungs, liver, and kidneys (Fig. 3). The third was extensive distribution of multiple lesions that could not be interpreted as common metastasis of malignancies. The last was rapid and significant response to steroid-based treatment.

**Table 2:** Summary of the image characteristics that form the pattern of IgG4-related disease on ^18^F-FDG PET/CT

Image characteristics	Confidence for indication of IgG4-RD
1. Diffusely elevated ^18^F-FDG uptake in organs, mainly involving salivary glands, pancreas, and prostate	
(1) Evenly, symmetrically distributed ^18^F-FDG uptake in the salivary glands without signs of infection	Strong
(2) Diffusely enlarged pancreas with moderate to intense ^18^F-FDG uptake without pancreaticobiliary duct obstruction	Strong
(3) Diffusely enlarged prostate with moderate to intense ^18^F-FDG uptake	Moderate
(4) Broadly involved lymph nodes with moderate to intense ^18^F-FDG uptake	Moderate
2. Patchy ^18^F-FDG-avid lesion without signs of infection, mainly involving aorta wall, retroperitoneal region, pancreas, bile duct, liver, kidney, and lung	
(5) Patchy thickness of aorta wall with moderate to intense ^18^F-FDG uptake not limited to the vascular intima	Strong
(6) Patchy retroperitoneal lesion with moderate to intense ^18^F-FDG uptake	Strong
(7) Patchy pancreatic lesion	Moderate
(8) Patchy bile duct lesion	Moderate
(9) Patchy liver lesion	Moderate
(10) Patchy lesions in the enlarged irregular kidneys	Moderate
(11) Patchy lung lesion	Weak
(12) Patchy pleural lesion	Weak
(13) Patchy pericardial lesion	Weak
3. Multi-organ involvement, including the following characteristics besides the above-mentioned	
(14) Pancreas nodule or mass	Weak
(15) Kidney nodule or mass	Weak
(16) Lung nodule(s)	Weak
4. Rapid, significant response to steroid-based treatment	
(17) The ^18^F-FDG-avid lesions had more than 80 % decrease of activity after 2 to 4 weeks of steroid-based treatment at a dosage of 40 mg to 50 mg prednisone per day	Strong

### Guiding biopsy and intervention

In seven cases, the new findings on ^18^F-FDG PET/CT resulted in the reselection of biopsy site to more accessible lesions, such as peripheral lymph nodes and submandibular gland. Figure 2 shows one of the biopsy results with typical inflammatory-cell infiltration consisting of a large amount of IgG4-positive plasma cells.

^18^F-FDG PET/CT also showed acute ureteral obstruction in five of the 12 patients with retroperitoneal fibrosis. The complete obstruction was characterized by hydronephrosis without radioactivity in the urine, whereas the partial obstruction had intense radioactivity in the urine retention. These findings opened the opportunity for timely recanalization of the ureters to avoid irreversible renal failure (Fig. 3).

### Evaluating treatment response

**Fig. 4:**
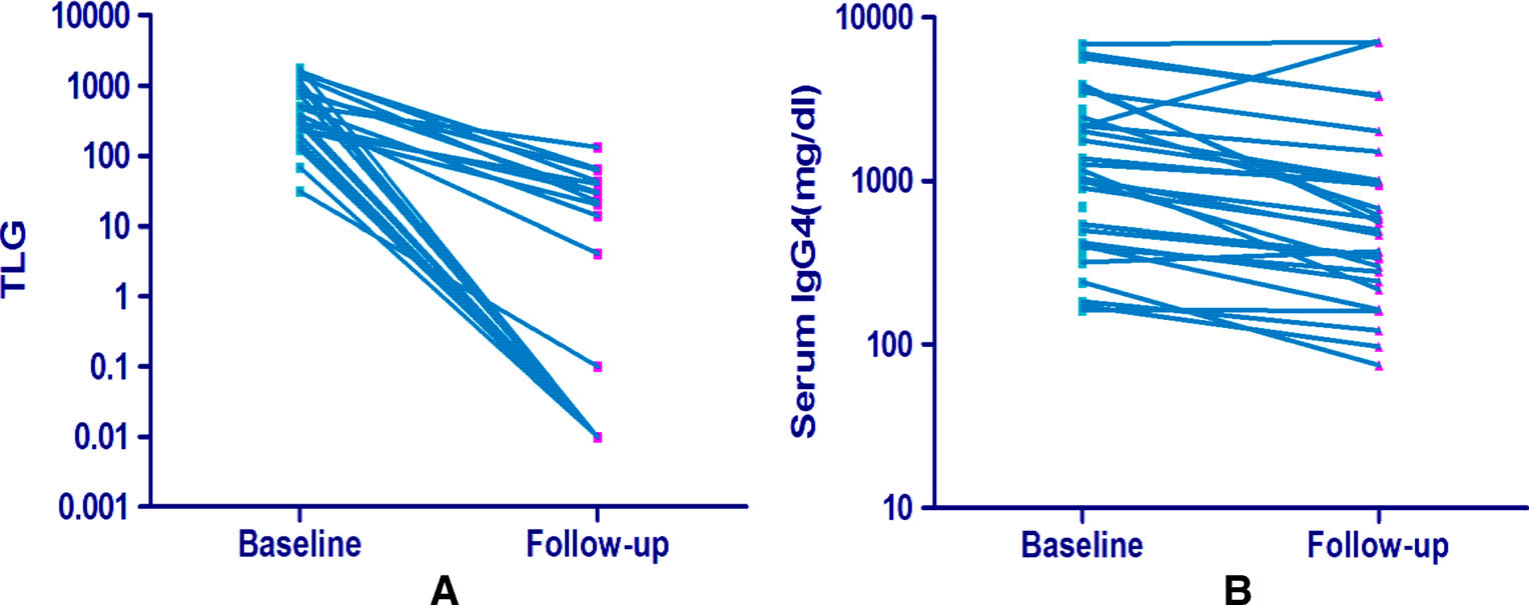
Changes in total lesion glycolysis (TLG) demonstrated by ^18^F-FDG PET/CT (**a**) were more remarkable than the changes in serum IgG4 level (**b**) after 2 to 4 weeks of steroid-based treatment

All 29 cases that underwent PET/CT follow-ups 2 to 4 weeks after treatment showed a remarkable decrease in ^18^F-FDG uptake following steroid-based therapy at a dosage of 40 mg to 50 mg prednisone per day. A PET VCAR analysis reported complete metabolic remission in 21 cases (72 %), whereas the other eight cases showed partial metabolic response with an 81.8 % to 98.9 % (89.0 % ± 6.4 %) decrease in TLG. Clinical symptoms and signs of the patients, including the allergies, were significantly relieved. Serum IgG4 levels decreased only in 88.6 % (31/35) cases and with less significance (Fig. 4). No significant correlation was found between the TLG and the IgG4 level in these patients (*r* = 0.37, *P* = 0.06).

## Discussion

Many questions about IgG4-RD, an emerging clinical entity most probably mediated by immune reaction, remain unresolved [1–3]. First of all, the diagnosis of IgG4-RD remains a challenge in clinical practice. In 2011, comprehensive diagnostic criteria for IgG4-RD were proposed, in which an abundant IgG4 positive cell infiltration (> 40 % of IgG positive plasma cells being IgG4 positive and > 10 IgG4 positive cells per high power field) in biopsy sample and an elevated serum IgG4 level over 135 mg/ml were emphasized [26]. These statements represent a major progress in the diagnosis of IgG4-RD. However, biopsy specimens are sometimes difficult to obtain and not always adequate for diagnosis [29]; serum IgG4 levels may also be normal in some patients with IgG4-RD, whereas an elevated serum IgG4 level can be encountered in a wide array of disorders, including allergic diseases, Castleman’s disease, and lymphoma [30–32].

In addition to the histopathological diagnosis and serological findings, IgG4-RD is also characterized by multi-organ involvements with special manifestations [1–3]. The disease has been recognized as a systemic immune-mediated inflammatory condition since 2003, when extrapancreatic lesions were identified with infiltration of IgG4-positive plasma cells in patients with AIP [7, 8]. Since then, IgG4-RD has been found in almost every organ [9–13]. Therefore, we believe non-invasive imaging characteristics should also serve as an important tool in the diagnosis of IgG4-RD, and a systematic, comprehensive understanding of the distribution information of IgG4-RD is vital for its diagnosis, staging, and treatment. However, conventional imaging methods, such as ultrasonography, CT, and magnetic resonance imaging (MRI), have a limited value in the provision of a comprehensive evaluation of the disease, although some characteristics of diffuse and focal infiltration are considered specific in some organs, such as the pancreas [33]. Whereas ^18^F-FDG PET/CT, which holds an advantage in providing metabolic information of the whole body in one scan, may be a valuable tool to evaluate the systemic inflammatory disease and reflect the involvements in each organ and the activity in each lesion, as well [16, 17].

Recently, several case reports and a retrospective analysis have preliminarily indicated a possible value of ^18^F-FDG PET/CT in evaluation of patients with IgG4-RD [18–25]. Most of these reports focused on the differentiation of AIP from pancreatic cancer. Although there was no significant difference of pancreatic ^18^F-FDG uptake between patients with AIP and patients with pancreatic cancer, certain patterns, including multiple foci in the pancreas, ^18^F-FDG uptake of the hilar lymph node, and extrapancreatic uptakes, were believed to be indications of AIP [24, 25]. A recent retrospective analysis also showed that multi-organ involvement was found in 84.2 % (16/19) patients [18]. In our prospective study with a relatively larger cohort of patients, multi-organ/tissue involvement was found in 97.1 % (34/35) patients; ^18^F-FDG PET/CT detected significantly more lesions in 71.4 % of the patients than the conventional imaging methods; and 90 % patients enrolled with single organ disease were finally found with multi-organ involvement. Therefore, our study confirms that ^18^F-FDG PET/CT can define more lesions than conventional imaging methods by providing a whole-body metabolic condition and morphological abnormality; thereby, it can serve as a sensitive tool in assessing organ involvement and disease distribution.

Based on this prospective study, we further analyzed the imaging characteristics of ^18^F-FDG PET/CT in IgG4-RD patients by comparing them with our routine patients (mostly with malignancies). As listed in Table 2, the strongest indications that we believe establish an IgG4-RD diagnosis include: 1) evenly, symmetrically distributed ^18^F-FDG uptake in the salivary glands without signs of infection; 2) diffusely enlarged pancreas with moderate to intense ^18^F-FDG uptake without pancreaticobiliary duct obstruction; 3) patchy thickness of aorta wall with moderate to intense ^18^F-FDG uptake not limited to the vascular intima; 4) patchy retroperitoneal lesion with moderate to intense ^18^F-FDG uptake; and 5) the ^18^F-FDG-avid lesions had more than 80 % decrease of activity after 2 to 4 weeks of steroid-based treatment at a dosage of 40 mg to 50 mg prednisone per day. Although none of these characteristics alone is specific enough for diagnosis of IgG4-RD, co-existence of several characteristics simultaneously may strongly indicate the disease.

Another possible contribution of ^18^F-FDG PET/CT in diagnosis of IgG4-RD is its value in helping the selection of a minimal and adequate biopsy site [21, 34]. In contrast to conventional imaging techniques, ^18^F-FDG PET/CT reflects glucose metabolism and provides disease activity of lesions. Therefore, ^18^F-FDG PET/CT can be a useful tool to localize an appropriate biopsy site, especially in cases with a negative result using conventional imaging methods, or those with multiple lesions but without functional preference. In our study, biopsy was performed in 20 patients. Among them, new findings of ^18^F-FDG PET/CT resulted in reselection of biopsy sites in seven patients. Here, we believe that ^18^F-FDG PET/CT has played an important role in selection of biopsy site, and further infer that a PET/CT guided biopsy will decrease the false-negative rate by providing a more accessible lesion with high metabolic activity.

Although there is no standardized therapeutic protocol for IgG4-RD, several points about treatment are clear. Without a fully established evaluation system, it is hard to outline the severity of the disease in an individual patient. Therefore, the intensity of a treatment is based on experience, mainly according to the clinical manifestations rather than the severity and extent of the disease. Steroid-based therapy is considered as the first-line therapy to both pancreatic and extrapancreatic lesions, and seems to be effective in the majority of the patients [35]. When vital organs are involved or life-threatening complications happen, aggressive treatment is need. For example, in patients with aorta involvement, the IgG4-related aortitis can finally lead to aortic aneurysms, which is a lethal condition, and a surgical procedure may be required [36, 37]. However, aortitis is usually diagnosed when severe complications occur, since it is asymptomatic at the early stage when it can be treated simply by using steroids. In our study, none of the patients had been found with aorta involvement before the ^18^F-FDG PET/CT scan; however, eight patients were proved to have aortic involvement by ^18^F-FDG PET/CT scans. Moreover, ^18^F-FDG PET/CT showed acute ureteral obstruction in five of the 12 patients with retroperitoneal fibrosis, which allowed opportunities for recanalization of the ureters to avoid irreversible renal failure. Therefore, our study showed that ^18^F-FDG PET/CT could be a useful tool in unsealing the insidious conditions that might lead to severe complications at the early stage.

On the other hand, there is still no standardized method to evaluate the activity of the disease and response to treatment. Laboratory tests, such as elevated serum IgG4 or IgG, complement consumption, erythrocyte sedimentation rate, and recently, the B cell-activating factor of the tumor necrosis factor family (BAFF) and a proliferation-inducing ligand (APRIL) , together with imaging findings, are considered to be associated with disease activity [35, 38, 39]. However, as mentioned above, some serum markers such as IgG4 level may be normal in some patients, and conventional imaging methods also have limitations in monitoring the disease since they only reflect morphological features. Recently, a responder index for IgG4-RD was proposed, which emphasized the subjective evaluation of lesions activity as well as serum IgG4 level [40]. Although this effort has made it easier for physicians to make a comprehensive evaluation, there is still a challenge to monitor the response to treatment. In our prospective study, most of the 29 patients who underwent follow-up ^18^F-FDG PET/CT had an early, complete metabolic response to steroid-based therapy, which was in accordance with other reports [18, 23, 38]. In addition, we further analyzed the changes between the baseline and follow-up scans, and classified response levels according to the PET Response Evaluation Criteria in Solid Tumor (PERCIST, version 1.0). With only 2 to 4 weeks of steroid-based treatment, 72.4 % (21/29) patients showed complete remission and the others also exhibited a ≥ 81.8 % decrease of TLG, whereas the serum IgG4 levels decreased in only 88.6 % cases and with much less significance. The prompt, quantitative response information provided by ^18^F-FDG PET/CT may be useful for evaluation of new treatment methods for IgG4-RD [41], although further studies are needed to exclude possible overestimation of therapeutic response. The early, rapid response to steroid-based treatment can also be used to verify the diagnosis and exclude possible concurrent malignancies [42, 43].

So far, ^18^F-FDG PET/CT has not achieved its rightful place in the consensus diagnostic criteria for IgG4-RD [26]. The above-mentioned data suggest that ^18^F-FDG PET/CT is an essential tool in many aspects of the diagnosis and treatment of IgG4-RD. To the best of our knowledge, this work is the first prospective study that uses a relatively large cohort of IgG4-RD patients to comprehensively clarify the usefulness of ^18^F-FDG PET/CT in evaluating multiorgan involvement, monitoring treatment response, and guiding biopsy and interventions.

The main limitation of this study is that only definitely diagnosed IgG4-RD cases were included in the analysis. As shown in Table 1, most patients in this group presented with Mikulicz’s disease (40.0 %), autoimmune pancreatitis (34.3 %), or retroperitoneal fibrosis (22.9 %). Such conditions might result in selection bias, because these kinds of patients were more easily diagnosed to have IgG4-RD and readily enrolled, whereas the rare or complicated conditions of IgG4-RD, such as those with normal IgG4 levels, were excluded. Therefore, this study represents a preliminary investigation of the characteristics of IgG4-RD using ^18^F-FDG PET/CT. In future studies, patients suspected to have IgG4-RD, especially those with malignancies, such as lymphoma with elevated serum IgG4 levels, should be included to determine the diagnostic accuracy of ^18^F-FDG PET/CT in IgG4-RD.

## Conclusions

In conclusion, whole body ^18^F-FDG PET/CT can provide a comprehensive view of the organs/tissues involved in IgG4-RD, and detect a larger number of lesions than conventional imaging methods such as ultrasonography and CT. The image characteristics or pattern of IgG4-RD observed on ^18^F-FDG PET/CT may be used for the indication or diagnosis of IgG4-RD. ^18^F-FDG PET/CT also aids in the selection of biopsy site and guiding the recanalization of ureteral obstruction. Moreover, this method is valuable for early response monitoring to achieve personalized treatment of the disease. A rapid, significant response to steroid-based therapy may also help verify the diagnosis and exclude possible malignancies. Therefore, ^18^F-FDG PET/CT is a useful tool that has a potential to uncover more details and provide a better understanding of IgG4-RD. Further confirmation in patients suspected to have IgG4-RD is needed, and when proven useful in differentiation with malignancies, especially lymphoma, these image characteristics of ^18^F-FDG PET/CT should be updated into the consensus diagnostic criteria for IgG4-RD.
